# Liver X receptor agonist upregulates LPCAT3 in human aortic endothelial cells

**DOI:** 10.3389/fphys.2024.1388404

**Published:** 2024-04-17

**Authors:** Delphine Bousquet, Elie Nader, Philippe Connes, Nicolas Guillot

**Affiliations:** ^1^ University Lyon, LIBM EA7424, Vascular Biology and Red Blood Cell Team, Universite Lyon 1, Villeurbanne, France; ^2^ Labex GR-Ex, PRES Sorbonne, Paris, France; ^3^ INSA Lyon, Villeurbanne, France

**Keywords:** endothelial cells, LXRs, LPCAT, polyunsaturated fatty acid, metabolism, cell signaling, endothelium

## Abstract

**Objective:**

Endothelial cells (ECs) play an important role in tissue homeostasis. Recently, EC lipid metabolism has emerged as a regulator of EC function. The liver X receptors (LXRs) are involved in the transcriptional regulation of genes involved in lipid metabolism and have been identified as a potential target in cardiovascular disease. We aimed to decipher the role of LXRs in the regulation of lipid metabolism in human aortic endothelial cells.

**Approach and Results:**

Lipid composition analysis of endothelial cells treated with the LXR agonist T0901317 revealed that LXR activation increased the proportion of polyunsaturated fatty acids (PUFAs) and decreased the proportion of saturated fatty acids. The LXR agonist decreased the uptake of fatty acids (FAs) by ECs. This effect was abolished by LXRα silencing. LXR activation increased the activity and the expression of lysophosphatidylcholine acyltransferase, LPCAT3, which is involved in the turnover of FAs at the sn-2 position of phospholipids. Transcriptomic analysis also revealed that LXRs increased the expression of key genes involved in the synthesis of PUFAs, including FA desaturase one and 2, FA elongase 5 and fatty acid synthase. Subsequently, the LXR agonist increased PUFA synthesis and enhanced arachidonic acid, eicosapentaenoic acid, and docosahexaenoic acid content in the EC phospholipids. Modification of the FA composition of ECs by LXRs led to a decrease of arachidonate and linoleate derived prostaglandins synthesis and release. No change on markers of inflammation induced by plasma from sickle cell patient were observed in presence of LXR agonist.

**Conclusion:**

These results identify LXR as a key regulator of lipid metabolism in human aortic endothelial cells and a direct effect of LXR agonist on lysophosphatidylacyl transferase (LPCAT3).

## Introduction

Endothelial dysfunction is one of the first steps in the development of cardiovascular disease (Vanhoutte et al., 2017). Endothelial cells (ECs) are able to regulate platelet aggregation, leukocyte interactions and vascular tone by releasing a variety of factors, including lipid mediators such as prostaglandins and eicosanoids (Ricciotti and FitzGerald, 2011; Godo and Shimokawa, 2017). These lipids derive from fatty acids (FA) contained in the membrane phospholipids (PLs) of the cells suggesting that PLs serve as a reservoir of substrate for the generation of bioactive lipids (Wymann and Schneiter, 2008; Ricciotti and FitzGerald, 2011). Moreover, deformability of ECs during angiogenesis is impacted by the FAs composition of the membrane (Ayee et al., 2017). Indeed, FA composition of the EC membrane is a major determinant of the biological properties (Caires et al., 2017) and function of ECs. However, the precise mechanisms controlling the FA distribution and metabolism in ECs remain poorly understood.

PLs are composed of one hydrophilic head group at the *sn*-3 position, a saturated fatty acyl chain at the *sn-*1 position and a saturated, mono or poly unsaturated fatty acyl chain at the *sn-*2 position of the glycerol backbone (Takamori et al., 2006; Wymann and Schneiter, 2008). Length and number of unsaturation determine the biophysical properties of the membranes, which in turn determine the membrane-associated cellular processes. It has been shown that PLs containing polyunsaturated FAs (PUFAs) allow the membrane to be more flexible, then reducing the energy cost required for cell deformation, which would be important during angiogenesis since this process require from ECs a highly plastic membrane to switch from a quiescent to a proliferative state (Pinot et al., 2014; Wang and Tontonoz, 2018a). PUFAs are also important for ECs signaling function as they are the source of bioactive lipid mediators. Among them the prostacyclin, PGi2, is the most potent endogenous inhibitor of platelet aggregation and is formed when the PUFA arachidonic acid (AA, C20:4n-6) is released from PLs by phospholipases and metabolized by the sequential actions of cyclooxygenase and prostacyclin synthase (Moncada et al., 1976; Ricciotti and FitzGerald, 2011). PLs are synthesized via the Kennedy pathway, however the absence of specificity from the enzyme involved in this reaction would not allow the highly diverse and asymmetrical distribution of fatty acyls chain that characterized the PLs (Wang and Tontonoz, 2019). Therefore the composition of PLs is mainly dependent on the activity of enzymes involved in the Land’s cycle through a process of deacylation and reacylation reactions (Lands, 1958). However, little is known about the regulation of this process in endothelial cells.

Phosphatidylcholine (PC) is the most abundant glycerophophoslipids in membrane cells (Van Meer et al., 2008) and the turnover of FAs at the sn-2 position of PCs is controlled by the actions of phopholipases A2 and lyso-PC acyltransferase (LPCAT). PUFAs are preferentially incorporated into phosphatidylcholine by the lyso-PC acyltransferase 3 (LPCAT3). Over the past decade, studies have highlighted the role of LPCAT3 in some diseases, such as atherosclerosis. LPCAT3 is expressed in small intestine, liver, adipose tissue and kidney where it regulates PLs metabolism. LPCAT3 has been involved in the secretion of very low density lipoprotein in mice (Li et al., 2012). Specific deletion of LPCAT3 in the small intestine result in hypoglycemia and reduced postnatal survival (Li et al., 2012; Rong et al., 2015). It has been shown that LPCAT3 gene promoter contains a liver X response element (LXRE), which is regulated by LXR agonist at least in chicken and human hepatoma cells (Demeure et al., 2011). Regulation of LPCAT3 expression by LXR agonist has also been demonstrated in macrophages and human monocytes and is involved in atherosclerosis development (Ishibashi et al., 2013; Thomas et al., 2018). Activation of LPCAT3 in human macrophages is responsible for an accumulation of AA in PLs and enhances secretion of AA-derived eicosanoids PGE2 and thromboxane A2 (Ishibashi et al., 2013). LPCAT3 knock out in macrophages promotes atherosclerosis development in Ldlr^−/−^ mice, which was associated with the increase of monocyte count (Thomas et al., 2018). However, the role of LPCAT3, if any, in ECs is unknown.

Recent studies have also highlighted that FA metabolism may regulate ECs function. Indeed, ECs are able to regulate the expression of FA transporters, such as CD36 and fatty acid transporter proteins (FATPs), which leads to the prevention of saturated FA-induced lipotoxicity (Schwenk et al., 2010). Fatty acid synthase (FASN) mediates *de novo* FAs synthesis and the loss of FASN in the endothelium impairs angiogenesis (Bruning et al., 2018). FAs are used in ECs as substrates for energy generation through FA β-oxidation (FAO), and inhibition of this reaction impairs nucleotide synthesis for endothelium DNA replication (Schoors et al., 2015). Moreover, endothelial deficiency of carnitine palmitoyltransferase (CPT)1A, the rate limiting enzyme in FAO, reduces EC proliferation (Schoors et al., 2015). However, the mechanisms involved in the regulation of FA metabolism in ECs are not defined.

Liver X receptors (LXRs) α and β are members of the nuclear receptor superfamily that function as ligand-dependent transcription factors. In the liver, the roles played by LXRs as activators of reverse cholesterol transport have been well described. The activity of the LXRs has also been well characterized in macrophages, where they are involved in the synthesis of the monounsaturated fatty acids (MUFAs) and PUFAs, by activating the expression of key lipogenic genes. Furthermore, LXRs activation in macrophages stimulates LPCAT3 activity, resulting in the incorporation of AA into the polar lipid fraction (Ishibashi et al., 2013). While the roles of LXRs are well defined in liver and macrophages, little is known about their role in ECs. Nevertheless, it has been shown that LXRβ can stimulate angiogenesis by interacting with the lipid raft domains of the EC membranes (Noghero et al., 2012; Ishikawa et al., 2013). Oxysterols, lipid-derived LXR agonists, have been shown to increase endothelium-leukocyte interactions and synthetic LXR agonist (T0901317) decreases, in some conditions, the expression of the inflammatory markers ICAM-1 and VCAM-1 (Morello et al., 2009). Considering the ability of LXRs to regulate FA metabolism in macrophages and the liver and given the importance of FA metabolism in EC function, it seems possible that LXRs could have an impact on FA metabolism in ECs and could modulate membrane composition via the activity of the LPCAT3 enzyme.

## Methods

### Cell culture

Human aortic endothelial cells (HAOEC) were purchased from Promocell (Heidelberg, Germany), and cultured in MV2 endothelial cell growth medium at 37°C and with 5% CO2. At least 4 independents batch of cells have been used in these experiments. The cells were not cultured above the fifth passage. HAOEC were transfected with LXRα or scramble siRNA (Silencer selected pre-designed siRNA, Ambion, Thermofisher scientific) using Lipofectamine RNAiMAX (Thermofisher scientific), as previously described (Guillot et al., 2012). The LXRs agonist, T0901317, was purchased from Cayman chemical.

### Fatty acid profile analysis

HAOEC were treated with T0901317 at the indicate concentration or vehicle (DMSO) for the indicated time and then washed. Lipids were then extracted from cell pellets using the Blight and Dyer method (Bligh and Dyer, 1979). Neutral lipids, free fatty acids and polar lipids were isolated and purified using aminopropyl bonded phase columns. The fatty acid composition of each fraction was then determined by gas-chromatography mass spectrometry (GC-MS) as described in details (Guillot et al., 2009; Ishibashi et al., 2013).

### Palmitate uptake

HAOEC were treated with T0901317 or vehicle (DMSO) then incubated with 2 µM of Bodipy FLC16 (Thermofisher scientific) for 15 min at 37°C. Cells were then washed, detached with accutase, and analyzed by flow cytometry using a BD Accuri C6 plus flow cytometer (BD Biosciences) according to the manufacturer instructions.

### LPCAT activity and cPLA2 activity

HAOEC were treated with T0901317 or vehicle (DMSO) then washed. Cell pellets were resuspended and lysed in Tris-HCl buffer (100 mM, pH 7.4, 200 mM Sucrose and 1 mM EDTA). Lysate was centrifugated for 5 min at 1000 g at 4°C to remove debris. 50 μg of cell protein extract was mixed with 100 mM Tris-HCl pH 7.4, 50 μM of Arachidonyl-CoA, 50 μM of Lysophsopatidycholine (LysoPC) and 1 mM of 5,5-dithio-bis-2-nitrobenzoic acid (DTNB) (Sigma Aldrich, Saint Quentin Fallavier). Arachadonic acid incorporation into LysoPC was followed in real-time for 10 min at 560 nm and 37°C, as previously described (Saunders et al., 2009). Activity of phospholipase A2 was measured using EnzChek Phospholipase A2 assay kit (Invitrogen) by fluorometric method according to the manufacturer’s protocol.

### Fatty acid β-oxidation (FAO)

HAOEC were submitted to shear stress or left under static condition in complete MV2 medium with 100 μM unlabeled palmitate and 50 μM carnitine. Cells were then incubated for 2 h in complete medium containing 2 μCi/mL [9,10 ^3^H] palmitate (Perkin Elmer, France). Medium was transferred into glass vial and [^3^H]-H_2_O was captured on a piece of Whatman paper soaked with H_2_O over a period of 48 h at 37°C. Radioactivity was measured by liquid scintillation counting (Schoors et al., 2015; Kalucka et al., 2018).

### RNA isolation and real time quantitative polymerase chain reaction

Total RNA was isolated from cultured cells using Tri Reagent solution (Thermofisher scientific) according to the manufacturer’s instructions. cDNA was synthesized from 1 μg of RNA using a PrimeScript RT reagent kit (Ozyme, Saint Quentin en Yvellines, France) and random hexamers (Thermo Scientific). Real time quantitative PCR was carried out using a Rotor-Gene Q (Qiagen, Hilden, Germany) and SYBR qPCR Premix Ex Taq (Tli RNaseH Plus) reagents. The results were normalized using TBP (TATA box binding protein) mRNA concentrations, measured as a reference gene in each sample.

### Prostaglandin and eicosanoid analyses

HAOEC were treated with T0901317 or vehicle (DMSO) for the indicated time. For extraction, each cell pellet was crushed with a FastPrep-24 Instrument (MP Biomedical) in 500 μL of HBSS (Invitrogen). After 2 crush cycles (6.5 m/s, 30 s), 10 µL were withdrawn for protein quantification. 300 μL of cold methanol (MetOH) and 5 µL of internal standard (Deuterium labeled compounds) were added. After centrifugation at 900 g, supernatants were transferred into 96-well deep plates, and diluted in H_2_O. Samples were then submitted to solid phase extraction (SPE) using an OASIS HLB 96-well plate (30 mg/well, Waters) that was pretreated with MetOH (1 mL) and equilibrated with 10% MetOH. After sample application, the extraction plate was washed with 10% MeOH (1 mL), and dried using aspiration. Lipid mediators were then eluted with 1 mL of MetOH. Prior to LC-MS/MS analysis, samples were evaporated using nitrogen gas, and reconstituted in 10 µL of MeOH.

LC-MS/MS analyses of eicosanoids were performed as previously described (Le Faouder et al., 2013). Briefly, lipid mediators were separated on a ZorBAX SB-C18 column (2.1 mm, 50 mm, 1.8 µm) (Agilent Technologies) using an Agilent 1,290 Infinity HPLC system (Technologies) coupled with an ESI-triple quadruple G6460 mass spectrometer (Agilent Technologies). Data were acquired in Multiple Reaction Monitoring (MRM) mode with optimized conditions (ion optics and collision energy). Peak detection, integration and quantitative analysis were done using Mass Hunter Quantitative analysis software (Agilent Technologies) based on calibration lines built with commercially available eicosanoid standards (Cayman Chemicals).

### PUFA biosynthesis

Linolenic acid-d_4_ and linoleic acid-d_4_ (Cayman chemical), 4 μM, were incubated with 0.8 mg/mL fatty acid free BSA in MV2 medium for 2 h at 37°C. HAOEC were treated for 24 h with T0901317 (10 μM) or vehicle (DMSO) then incubated with the deuterated fatty acid-BSA mix. Total lipids were extracted using the Folch method, and then analyzed and quantified by gas chromatography-mass spectrometry (GC-MS) negative chemical ionization (NCI) with a specific determination of corresponding synthetized n-6 deuterated fatty acids (C18:2 n-6 d4, C18:3 n-6 d4, C20:3 n-6 d4, C20:4 n-6 d4, C22:4 n-6 d4, C24:4 n-6 d4, C24:5 n-6 d4, C22:5 n-6 d4) and n-3 deuterated fatty acids (C18:3 n-3 d14, C18:4 n-3 d12, C20:4 n-3 d12, C20:5 n-3 d10, C22:5 n-3 d10, C24:5 n-3 d10, C24:6 n-3 d10, C22:6 n-3 d10) (Cayman chemical). Data were normalized to cell protein concentration.

### Plasma from healthy and sickle cell anemia patients

HAOEC were pre-treated with T0901317 (10 μM) or vehicle (DMSO) for 12 h and then incubated 4 h with plasma (5%) from sickle cell anemia patients (SS) or from ethnically matched healthy donors. All patients were in steady-state conditions, i.e., no blood transfusion in the previous 3 months and the absence of any acute complications for 1 month at least. Plasma samples were obtained from the DREPA-RHEOL protocol. This protocol was approved by the “Hospices Civils de Lyon–CPP Est” Ethics Committee (L14-127) and was performed in accordance with the guidelines set by the declaration of Helsinki.

### Flow cytometry staining

After treatment, cells were washed and detached with accutase (Thermofisher scientific). Cells were resuspended in PBS 0.5% BSA 2 mM EDTA and stained with VCAM-1, I-CAM1 or E-selectin antibody conjugated to fluorochromes (Thermofisher scientific). FACS analyses were carried out using a BD Accuri C6 Plus flow cytometer (BD Biosciences).

### Statistical analysis

Data are expressed as mean ± S.E.M. GraphPad Prism 6 software was used to assess statistical significance by using Kruskall-Wallis test and Mann- Whitney U test. A *p*-value of <0.05 was considered statistically significant.

## Results

### Activation of LXRs increases MUFA and PUFA content in human aortic endothelial cells

**FIGURE 1:**
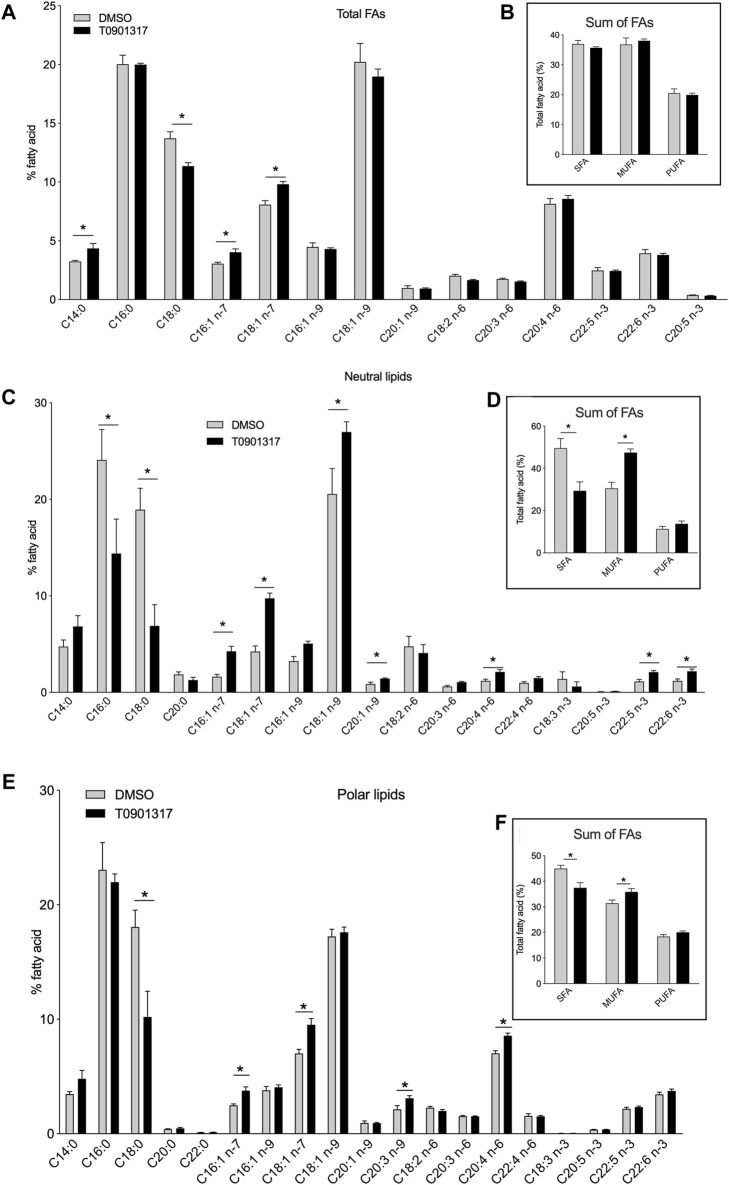
Liver X receptor agonist treatment changes fatty acid composition in human endothelial cells (ECs). Human aortic ECs were treated with T0901317 (10 μM, 48 h) or vehicle (DMSO). Relative proportions of individuals fatty acids in **(A)** the total fatty acid fraction, **(C)** the neutral lipid fraction, **(E)** the polar lipid fraction. Relative proportion of saturated, monosaturated (MUFA) and polyunsaturated fatty acid (PUFA) in **(B)** total lipid fraction, **(D)** neutral lipid fraction and **(F)** polar lipid fraction. Values are mean ± SEM of 4 independent experiments. *p < 0.05, Mann-Whitney test.

To elucidate the impact of LXRs activation on EC lipid profile, we treated cells with the LXRs agonist T901317. LXRs activation did not change the proportion of saturated fatty acid (SFAs), monounsaturated fatty acids (MUFAs) or polyunsaturated fatty acids (PUFAs) (Figure 1A,B). In total FAs fraction we observed an accumulation of C16:1n-7, C18:1n-7 as observed in neutral and polar lipids fraction. The sum of MUFAs was however not statistically different in total FAs fraction between LXR agonist treated cells and control cells. (Figure 1B). LXRs activation increased the content in C18:1n-7 and C16:1n-7 FA, while it decreased the concentration of stearic acid (C18:0). We also found that arachidonic acid was the most abundant PUFAs in human aortic endothelial cells (Figures 1A,B).

To gain insight into the FA analysis, we separated the polar and neutral lipid fractions from ECs treated with T0901317 or vehicle. Specific pattern of FAs was observed between polar and neutral FAs. In the neutral lipids fraction, LXRs activation significantly decreased the proportion of SFAs, especially stearic acid (C18:0) and palmitic acid (C16:0) (Figures 1C,D). In the meantime, we observed an increase of the relative amount of MUFAs C16:1n-7, C18:1n-7 and C18:1n-9 (Figures 1C,D). PUFAs levels were also significantly increased after LXRs activation. LXR agonist T0901317 increased arachidonic acid (C20:4n-6) and docosahexaenoic acid (DHA, C22:6n-3) levels in the neutral lipid fraction (Figures 1C,D). LXR activation induced the same effect on the polar lipid fraction that encompasses the glycerophospholipids (Figures 1E,F). As shown in Figures 1E,F, treatment with T0901317 resulted in a marked decrease of SFAs, and an increase of MUFAs in the polar lipid fraction. Arachidonic acid content was significantly increased after T0901317 treatment compared to vehicle. The effect of LXR was for some FAs related to the lipids fraction. C16:0 concentration decrease after LXR activation in neutral FAs fraction but not in the polar fraction, DHA and EPA concentration were not modulated in neutral fraction compared to polar one (Figures 1C,D). These results suggest that LXR pathway activation could significantly alter the lipid composition of ECs, specifically at the polar and neutral lipid fraction levels, with an increase of MUFAs, a specific increase of some PUFAs (arachidonic acid, EPA and DHA) and a decrease of SFAs content.

### Activation of LXRs decrease the uptake of FAs by ECs

**FIGURE 2:**
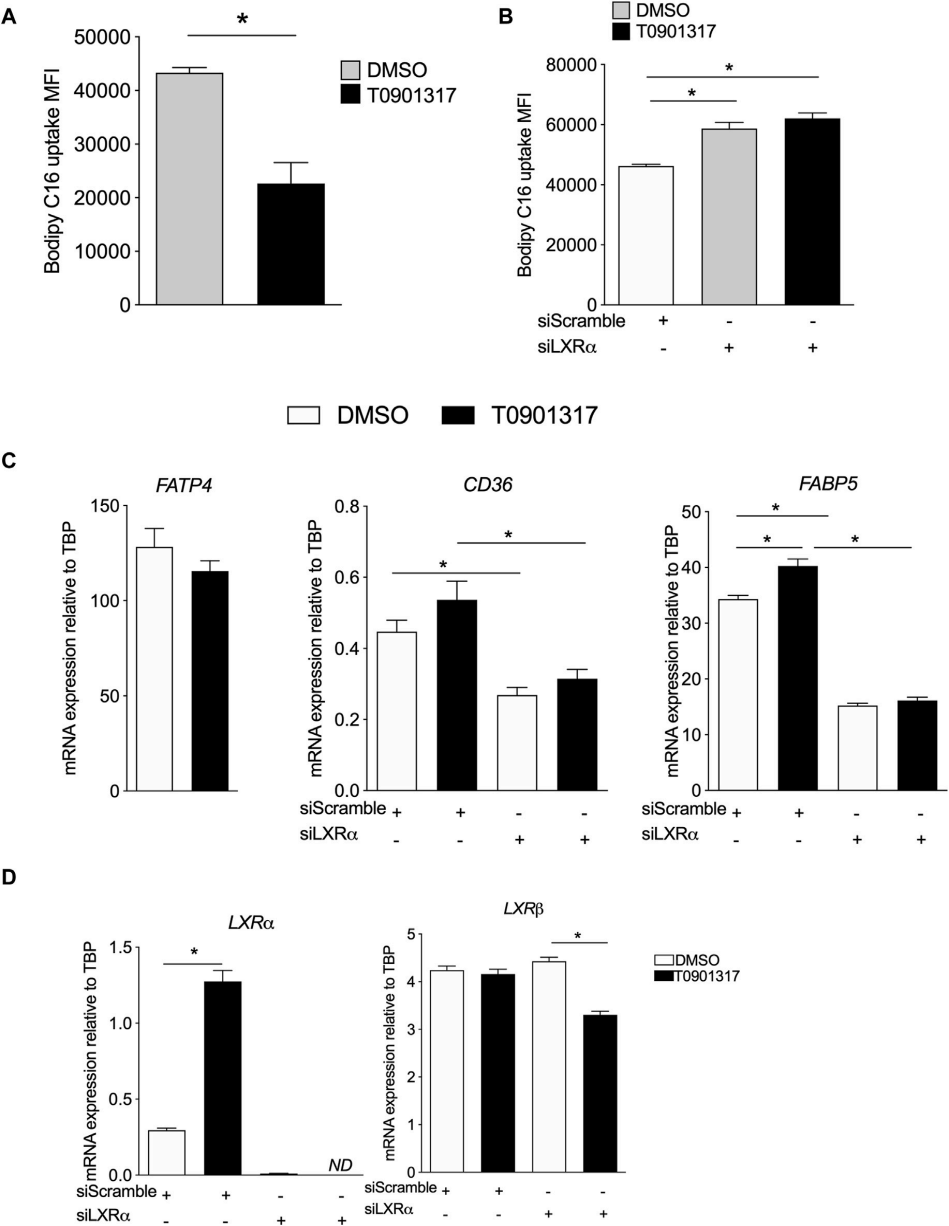
Liver X receptors agonist decreases Bodipy FLC16 uptake by ECs and modulate FA transport genes expression. **(A)** ECs were treated with T0901317 (10 μM, 24 h) or vehicle (DMSO), then washed and incubated in the presence of Bodipy FLC16, then analyzed by flow cytometry. **(B)** ECs were pretreated with siRNA scramble (siScramble) or siRNA LXRα then treated with T901317 as in **(A)**. Values are mean of fluorescent intensity (MFI) ± SEM of 4 independent experiments. **(C)** Relative mRNA expression of FABP5 and CD36 in ECs pretreated with siRNA scramble or siRNA LXRα then treated with T901317 or vehicle as in **(A)**. **(D)** expression of LXRα and β in ECs and confirmation of the invalidation by siRNA against LXRα. Values are mean ± SEM of 4-6 independent experiments. *p < 0.05, Mann-Whitney test.

We thus determined whether the observed changes in FA composition could be related to the capacity of ECs to take up FAs. ECs treated with T0901317 internalized significantly less palmitic acid probe compared to vehicle treated cells (Figure 2A), suggesting that LXR activation reduces palmitate uptake. Knock-down of LXRα increased the uptake of Bodipy FL C_16_ compared to siScramble ECs in the basal condition (Figure 2B). LXR activation did not decrease the uptake of FAs in ECs-siLXRα (Figure 2B), suggesting that LXRα activation inhibits FA uptake in ECs. We therefore analyzed whether LXR activation in ECs could modulate the expression of known fatty acid transport proteins, including fatty acid transport protein (FATP4), fatty acid binding protein (FABP5) and lipid-scavenger cell surface receptor CD36 (Hagberg et al., 2013). LXR activation had no effect on FATP4 mRNA expression (Figure 2C). However, compared to vehicle treated cells, T0901317 has no effect on CD36 mRNA expression (Figure 2C), and significantly increased the expression of FABP5 (Figure 2C). In basal conditions, knock-down of LXRα downregulated the expression of CD36 and FABP5 mRNA, suggesting that LXRs could modulate specifically the expression of FABP5 and CD36 mRNA. Indeed, the effect of T0901317 was abolished in siLXRα treated cells (Figure 2C), suggesting a pivotal role of LXRα in the control of CD36 and FABP5 expression. Knock-down of LXRα was confirmed by PCR (Figure 2D).

### LXRs activation increases LPCAT activity and expression in human aortic endothelial cells

**FIGURE 3:**
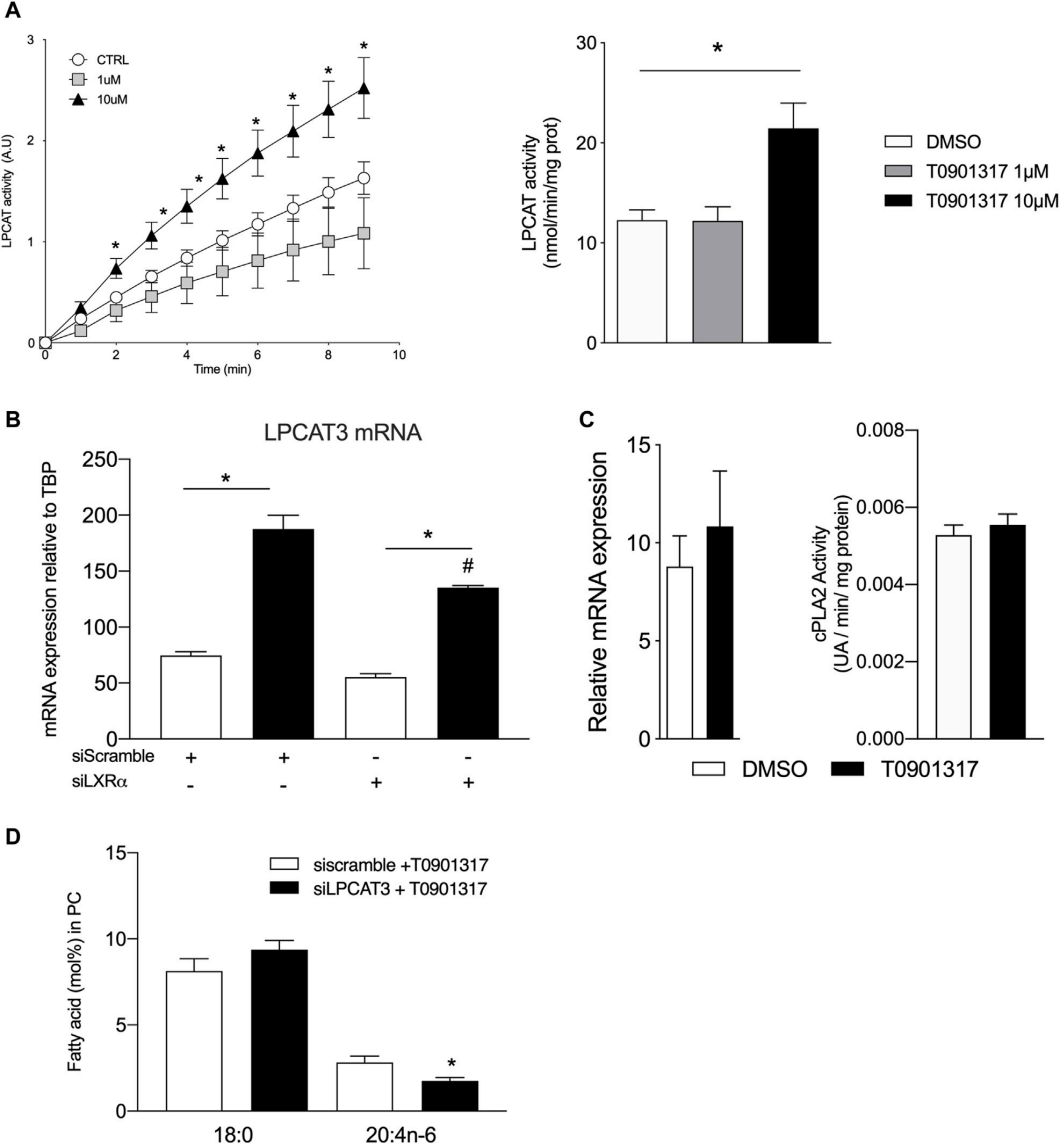
LXR agonist increases lysophosphatidylcholine acyltransferase LPCAT activity and expression in human endothelial cells. **(A)** LPCAT activity was measured by the spectrophotometric method using the reaction of thiol groups of the released CoA with 5,5-dithiobis (nitrobenzoic acid) in EC protein extracts treated with T0901317 or vehicle (DMSO). **(B)** Relative mRNA expression of LPCAT3 and **(C)** cPLA2 in ECs treated with T0901317 (10 µM) or vehicle. **(D)** 18:0 and 20:4n-6 content in ECs treated with LCPAT3 siRNA Data are mean ± SEM of 4–6 independent experiments. *p < 0.05 compared to specific control. #*p* < 0.05 compared to siScramble + T0901317, Mann-Whitney test.

We next hypothesized that LXRs might play a role in the regulation of enzymes that mediate the turnover of fatty acids at the *sn*-2 position of phospholipids. This reaction is controlled by the phospholipases A2 and lysophospholipid acyltransferases mainly the lysophosphatidylcholine acyl transferase 3 (LPCAT3). To test our hypothesis, we analyzed the expression and activity of the LPCAT3, which has been shown to be regulated by LXRs in monocytes (Demeure et al., 2011; Rong et al., 2013). The kinetic of LPCAT3 activity was analyzed using arachidonoyl-coA as substrate. The reaction measures the release of the coA group from the esterification of the arachidonoyl group to the *sn*-2 position of the lysophosphatidylcholine. As shown in Figure 3A, T0901317 significantly increased LPCAT3 activity, when used at 10 μM (Figure 3 A). LPCAT3 mRNA expression was also measured in parallel, and we observed that LXR agonist increased mRNA expression of LPCAT3 suggesting a role of LXR in LPCAT3 expression (Figure 3B). cPLA2 mRNA expression and PLA2 activity were not impacted by the treatment with LXR agonist (Figure 3C). These results suggest that the LXR agonist T0901317 increased LPCAT3 activity and mRNA expression in aortic endothelial cells and suggest a pivotal role of LXRs in PUFAs accumulation in the PLs membrane. Downregulation of LPCAT3 by siRNA significantly but modestly decreased the proportion of arachidonic acid in phosphatidylcholine suggesting a pivotal role of LPCAT3 in LXR mediated effect (Figure 3D).

### LXR activation stimulates the metabolism of fatty acid

**FIGURE 4:**
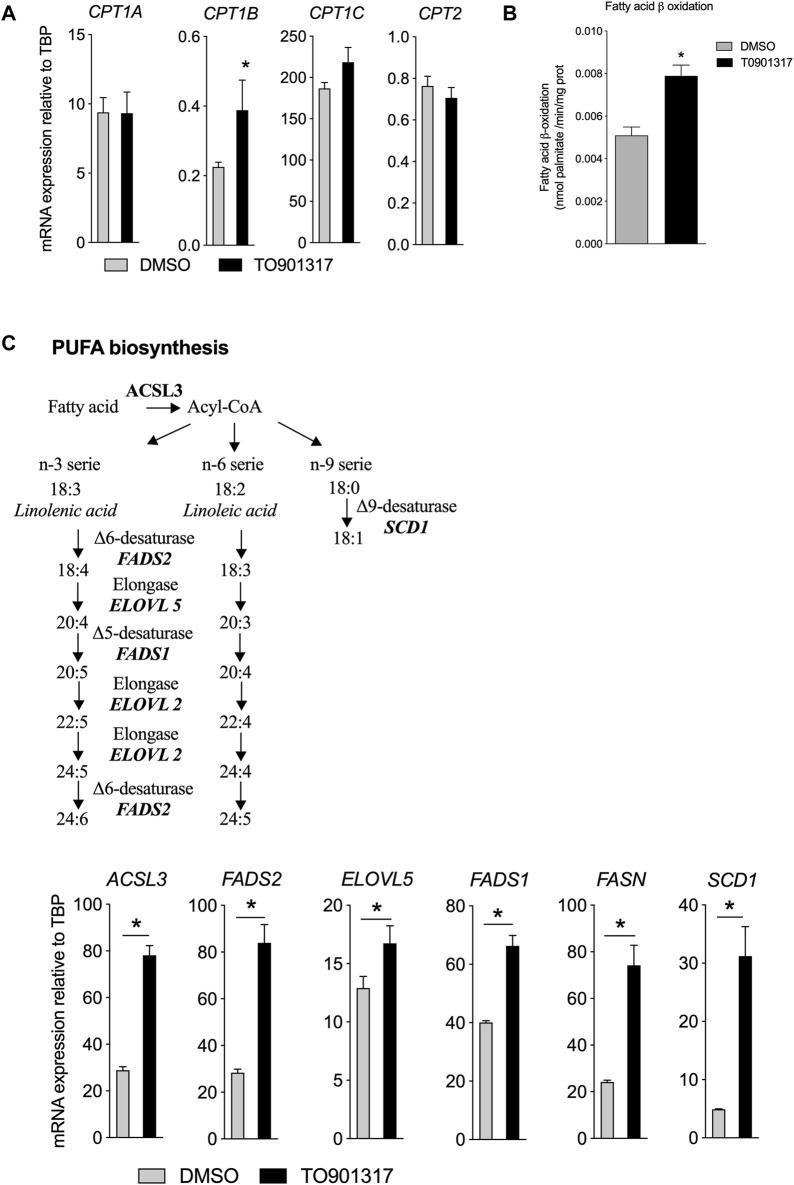
LXR activation increases fatty acid biosynthesis and upregulates related gene expression in ECs. ECs were treated by T0901317 (10 μM, 24 h) or vehicle (DMSO) and then RNA were extracted and analyzed. **(A)** gene expression related to fatty acid beat oxidation. **(B)** Fatty acid β oxidation measured in ECs and **(C)** Polyunsaturated fatty acids biosynthesis pathway and genes expression in ECs treated with T0901317. Data are mean ± SEM of 4–6 independent experiments. *p < 0.05, Mann-Whitney test.

The impact of LXR activation in FA uptake combined with the modulation of the cellular FA composition in ECs raised the possibility that LXRs may also contribute to the metabolism of FAs. Fatty acid β-oxidation (FAO) is critical for EC proliferation and redox homeostasis (Schoors et al., 2015) and we observed here that LXR activation increased significantly the expression of the rate limiting enzyme involved in FAO (CPT1B) (Figure 4A). No impact on CPT1 A, C and CPT2 of T0901317 was observed in our conditions (Figure 4A). To determine if change in mRNA expression would have an impact on FAO *per se* we measured the oxidation of FA after treatment of ECs with LXR agonist by the radioactivity method (Kalucka et al., 2018). We observed that T0901317 stimulated significantly the oxidation of [9,10 ^3^H] palmitate compared to control cells suggesting a direct role of LXRs in FAO flux (Figure 4B). Then, we examined the expression of genes related to *de novo* synthesis of MUFAs and PUFAs (Figure 4C). Overall, T0901317 treatment upregulated the expression of all the key genes involved in FA synthesis (Figure 4C). The acyl-coA synthase (ACSL3) that transforms the fatty acid to the corresponding acyl-coA ester was upregulated upon T0901317 treatment (Figure 4C). Fatty acyl-coA are then transformed by actions of delta-5 desaturase (FADS1 gene), delta-6 desaturase (encoded by FADS2 gene) and elongase (encoded by ELOVL5 gene) (Figure 4C). Expression of these genes were significantly increased by T0901317 (Figure 4C). Treatment of ECs with T0901317 also increased the expression of fatty acid synthase gene (FASN), which catalyzes the formation of palmitic acid (C16:0) from acetyl-CoA and malonyl-CoA, in the presence of NADPH (Figure 4C). Delta-9 desaturase gene (SCD1) was regulated by laminar shear stress. We showed that T0901317 significantly increased SCD1 mRNA levels (Figure 4C). Overall, these results suggest that LXR activation play a pivotal role in the turnover of the FA in human aortic endothelial cells.

**FIGURE 5:**
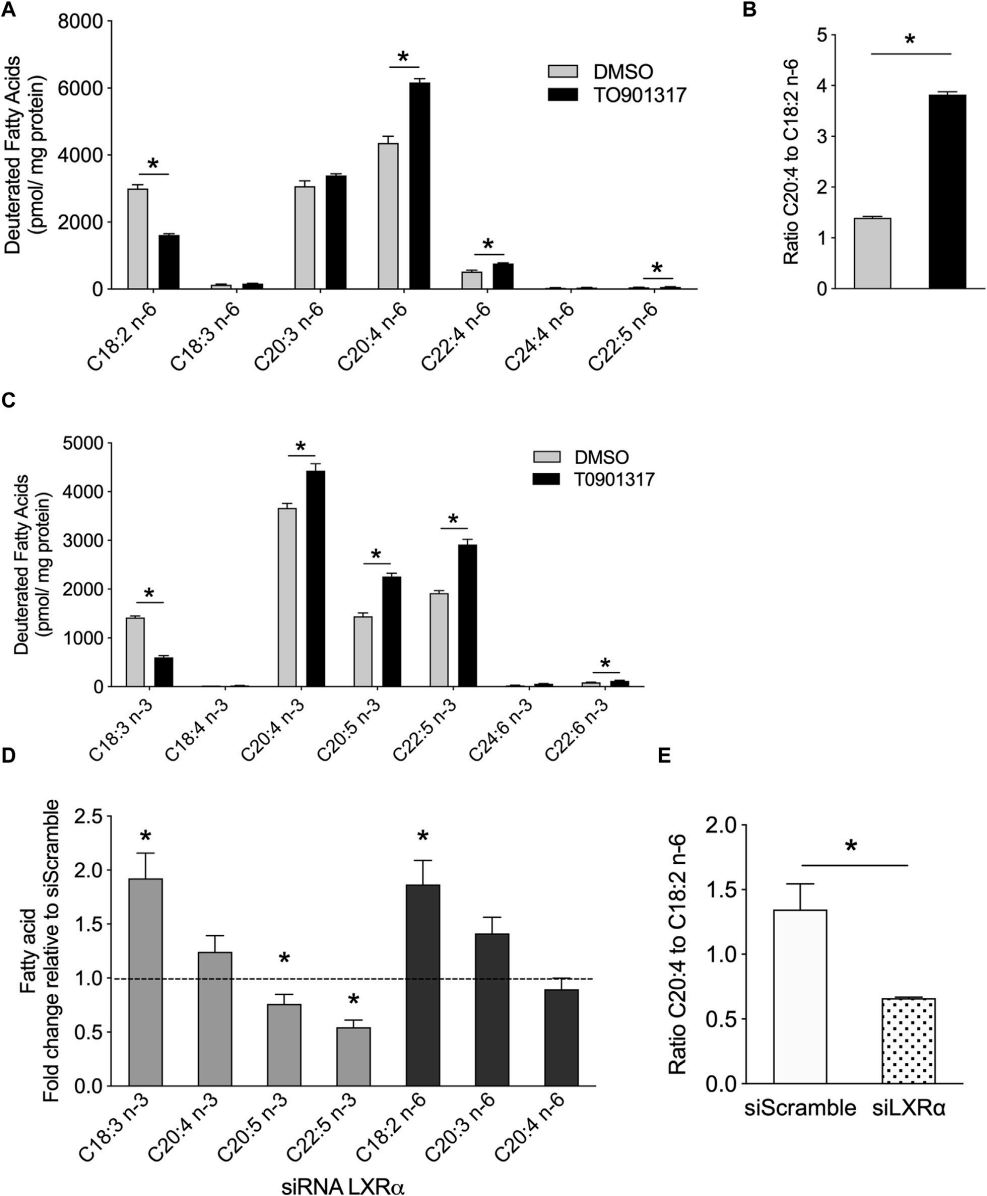
LXR agonist promotes n-6 and n-3 PUFA synthesis in human primary endothelial cells. ECs were treated with T0901317 (10uM) or vehicle (DMSO) for 24 h and then incubated with deuterated C18:2n-6 (linoleic acid) and C18:3n-3 (linolenic acid) for 24 h. Lipids were extracted and deuterated fatty acids were analyzed by GC-MS. **(A)** Amount of n-6 serie fatty acids. **(B)** Ratio of arachidonic acid to linoleic acid. **(C)** Amount of n-3 serie fatty acids. **(D)** Relative amount in basal condition of deuterated FAs in ECs pretreated with siRNA against LXRα or siRNA scramble. **(E)** ratio of arachidonic acid to linoleic acid in ECs siScramble and siLXRα. Data are represented as mean ± SEM of 4 independent experiments. *p < 0.05, Mann-Whitney test.

To gain insight the LXR function and to follow *in situ* the synthesis of MUFA and PUFA synthesis, ECs were pretreated with LXR agonist then treated with a tracer dose of deuterated linoleic acid (LA, C18:2n-6), precursor of n-6 PUFAs, and linolenic acid (ALA, C18:3n-3), the precursor of n-3 PUFAs. We observed that cells under LXR agonist treatment accumulated significantly more n-6 PUFAs (AA, C20:4n-6) and other long chain n-6 PUFAs (C22:4 and C22:5 n-6) than control cells (Figure 5A). Consequently, we observed a significant decrease of C18:2n-6 in treated cells, suggesting an upregulation of the conversion of C18:2n-6 to n-6 PUFAs upon LXR activation (Figure 5A). Furthermore, T0901317 increased the ratio AA:LA, which is an indirect measure of elongase and desaturase activity (Figure 5B), which confirmed the upregulation of n-6 PUFAs synthesis.

Long chain n-3 PUFA *de novo* synthesis shares the same enzymatic system with the n-6 PUFAs. Therefore, we looked at whether LXR activation could also increase the synthesis of n-3 PUFAs. LXR activation increased the conversion of C18:3n-3 to 20:4, 22:5 and 24:6 n-3. Most importantly, LXR agonist induced the accumulation of eicosapentaenoic acid (EPA, C20:5n-3), the precursor of E-series resolvins, and docosahexaenoic acid (DHA, C22:6n-3), the precursor of D-series resolvins (Serhan and Levy, 2018) (Figure 5C). Overall, these data show that LXR activation increased the conversion of C18:2n-6 to arachidonic acid and C18:3n-3 to EPA and DHA in ECs. To confirm the role of LXR in PUFA synthesis, we investigated the functional relevance of the LXRα deletion in PUFA synthesis. Knockout of LXRα induced the accumulation of C18:3n-3 and a significant reduction of C20:5n-3 and C22:5n-3 levels, suggesting that LXRs invalidation slowed down the synthesis of n-3 PUFAs from C18:3n-3. (Figure 5D). We also observed an inhibition of the conversion of linoleic acid in siLXRα treated cells compared to siScramble treated cells, thus leading to the accumulation of C18:2n-6 FA (Figure 5D). The ratio of arachidonic acid to linoleic acid was significantly lower in siLXRα ECs compared to siScramble ECs, suggesting overall, that LXRα promotes MUFAs and PUFAs biosynthesis in ECs (Figure 5E).

### Activation of LXRs decreases lipoxygenase derived lipid synthesis

**FIGURE 6:**
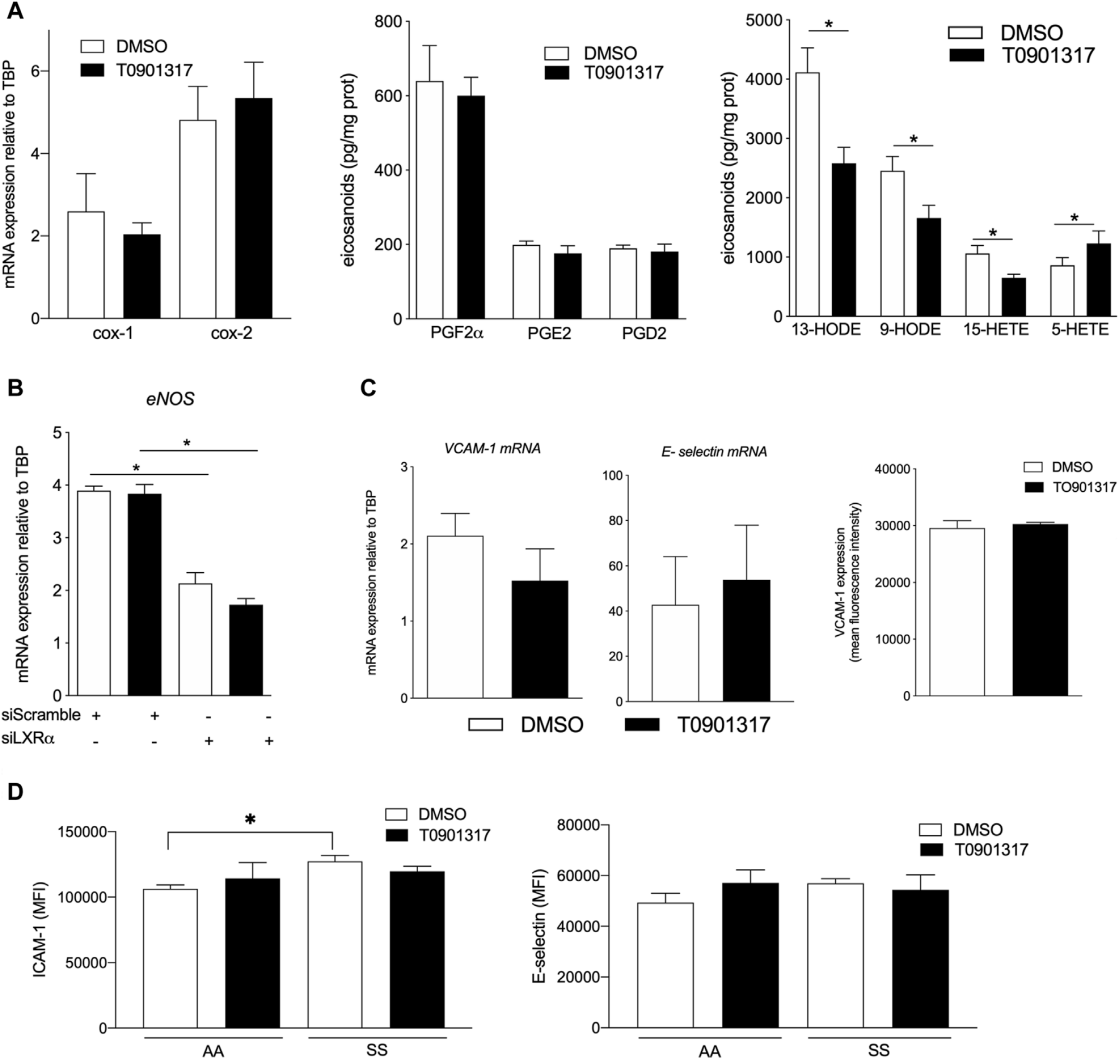
LXR agonist alters prostaglandin release from ECs. ECs were treated with T0901317 or vehicle (10uM for 24 h) and then cell pellets and medium were used to measure eicosanoid release in the basal condition. **(A)** lipids derived from cyclooxygenase and lipoxygenase activity. **(B)** eNOS mRNA expression relative to TBP in ECs treated with siRNA scramble or siRNA LXRα with T0901314 or vehicle. **(C)**, VCAM-1 and E-selectin mRNA expression measured by PCR and flow cytometry in ECs treated with T0901317 (10 µM) or vehicle and VCAM-1 expression measured by flow cytometry. **(D)**, ICAM-1 and E-selectin expression measured by flow cytometry in ECs treated with sickle cell anemia (SS) plasma or healthy plasma (AA) and T0901317 or vehicle. Data are represented as mean ± SEM of 5–6 independent experiments. *p < 0.05, Mann-Whitney test.

The synthesis and release of PUFA-derived prostaglandins from vascular endothelial cells has been shown to modulate vascular tone, platelet aggregation and inflammation (Ricciotti and FitzGerald, 2011). LXRs activation did not change the formation of PGF2α, PGE2 and PGD2 (Figure 6A). Interestingly, synthesis of lipoxygenase derived lipids was affected by LXR agonist treatment, as shown by the reduction of 13-HODE, 9-HODE, derived from linoleic acid, and 15-HETE, derived from arachidonic acid concentration after T0901317 treatment (Figure 6A). However, we observed an increase in 5-HETE derived from arachidonic acid through the 5 lipoxygenase activity. In parallel we looked at the expression of endothelial NO synthase and found that LXRα activation specifically regulated the expression of this enzyme (Figure 6B). Next, expression of VCAM-1 which is expressed during inflammation was not modulated by T0901317 at the mRNA level and at the surface membrane level. No modification was observed for E-selectin mRNA expression (Figure 6C). To assess further the role of LXR in an inflammatory disease model we analyzed the effect of plasma derived from sickle cell anemia patients, a disease characterized by chronic inflammation and where ECs dysfunction is well documented (Wagner and Frenette, 2008). ECs were pretreated with T0901317 and then treated with plasma from patients (SS) or volunteers (AA) and TNFα. As expected, I-CAM1 was upregulated in basal condition in SS plasma treated cells compared to AA plasma treated cells (Figure 6D) however LXRs activation did not change the expression of I-CAM1. Not effect of T0901317 was observed for E-selectin expression in ECs treated with AA plasma compared to SS plasma treated cells (Figure 6D). Overall activation of LXRs in ECs significantly modulated the release of PUFA-derived lipids and could also regulate the expression of eNOS.

## Discussion

Endothelial dysfunction is a key feature of vascular disease, such as atherosclerosis (Godo and Shimokawa, 2017), and FA metabolism has been shown to regulate endothelial function (Schoors et al., 2015). The fatty acyl chain composition of phospholipids determines the biophysical properties of endothelial cell membranes, and thereby affects angiogenesis and inflammation (Van Meer et al., 2008; Serhan and Levy, 2018). Furthermore, phospholipids are substrates for the generation of prostaglandins, the bioactive molecules involved in signal transduction (Van Meer et al., 2008). Previous studies have outlined the importance of LXRs in cholesterol metabolism, particularly in macrophages (Ishibashi et al., 2013; Varin et al., 2015), but little is known about LXRs and FA metabolism in endothelial cells. Here we demonstrate that LXRs activation could modulate lipid metabolism in human aortic endothelial cells. The accumulation of both MUFAs and PUFAs under LXRs activation, along with the decreased proportion of saturated FAs, was associated with a reduction of fatty acid uptake, which could be attenuated by blocking LXRα. Our results also show that LXR activation results in the upregulation of LPCAT3 activity which would lead to the accumulation of PUFAs in ECs membrane. Upregulation of the acylation reaction was associated with the increased synthesis of PUFAs from n-3 and n-6 precursors. Furthermore, while the activation of LXR activation seems to not have a significant impact on the expression of adhesion molecules, we observed that it could modulate the synthesis of some lipoxygenase derived lipids especially the 13-HODE, 9-HODE and 15-HETE. Our study adds to the current understanding of lipid metabolism in ECs by introducing a key role of LXRs. LXRs can control the uptake, the synthesis and therefore the phospholipid composition of ECs, and then contribute to the regulation of prostaglandins release.

Activation of LXRs in ECs produced a significant change in FAs composition of the ECs. Changes in cellular fatty acid composition may reflect an alteration in fatty acid transport across the cell membrane and, or in intracellular lipid trafficking (Wang and Tontonoz, 2018b). The first step was then to look at the transport of FAs and our results indicate that LXR pathway activation slows down the uptake of saturated FAs by ECs and siRNA-mediated LXRα knockdown could reverse this inhibition. Saturated FAs, such as palmitic acid, have been involved in modulating the expression of inflammatory protein (ICAM-1) (van Wanrooij et al., 2006). Therefore, the association between the decreased uptake of saturated FAs and the increased amount of PUFAs observed in our study may reflect a protective mechanism against palmitic acid -induced vascular endothelial dysfunction as suggested in a previous study (Ishida et al., 2015). Although fatty acids can move across the cell membrane by simple diffusion, there is a consensus that fatty acid-binding membrane proteins (FABPs) both facilitate and regulate the process of cellular fatty acid uptake (Glatz et al., 2010; Schwenk et al., 2010). Several fatty acid transporters have been identified, including CD36 and FABP5. Deficiencies in or malfunctioning of FABPs have been associated with lipid-related diseases, like atherosclerosis, in both humans and in animal disease models (Yu et al., 2015). Furthermore, FABP5 is involved in angiogenesis, as findings from a previous study have shown that its expression significantly enhanced EC proliferation, chemotactic migration, and angiogenic sprouting (Yu et al., 2015). FABP5 expression has been shown to be regulated by PPARδ, and FAs and eicosanoids have been identified as natural ligands of the PPARs (Yu et al., 2015). The accumulation of PUFAs observed in our study leading to an increase of potential ligands for PPARs could therefore explain the upregulation of PPARs target, such as FABP5. Further studies are needed to investigate the role of PPARs in LXRs agonist treated cells. FABP5 has also been shown to be involved in regulating the EC proliferation therefore suggesting a pivotal role for LXRs in ECs proliferation and angiogenesis as suggested by others studies (Walczak et al., 2004; Yu et al., 2015). FABP5 and CD36 mRNA expression were specifically regulated by LXRs with knock down of LXRα decreasing their expression. These data are consistent with analyses showing that CD36 is a target of LXRα(He et al., 2007). The function of the transporter CD36 in ECS is known as a potent negative regulator of angiogenesis (Febbraio et al., 2001) and our findings add a potent role yet to be evaluated in lipid metabolism. The results raise therefore the possibility that LXRs may have several targets to modulate the FAs fate and turnover in ECs.

LXR activation induced a significant change in FA composition of phospholipids but it is known that phospholipid composition is maintained through mechanisms of acylation and deacylation (Lands, 1958). Lysophosphatidylcholine acyltransferases (LPCAT) are a group of enzymes involved in the acylation reactions of fatty acyl chains into the sn-2 site of phosphatidylcholine. LPCAT3 belongs to the membrane-bound O-acyltransferase family, and has been involved in arachidonic acid distribution in human macrophages (Ishibashi et al., 2013). Here we show that LPCAT3 is abundant in human aortic endothelial cells and LXR activation increases the expression and activity of this enzyme. The upregulation of LPCAT3 mRNA expression could reflect the direct action of LXRs on the LPCAT3 promoter, which contains a functional LXR response element as observed on human hepatoma, monocytes and macrophages (Demeure et al., 2011). Our data also show an upregulation of LPCAT3 activity by LXR agonist that was directly related to the expression of LPCAT3 mRNA. However, the molecular mechanisms involved in this catalytic reaction remain to be identified. Studies have shown that LPCAT3 is involved in glycerophospholipid remodeling, which generates a local change in the membrane composition (Kazachkov et al., 2008; Shindou et al., 2008). Our data indicate that LXRs pathway activation increases arachidonic acid content in polar lipids and MUFA in neutral lipids. This is in accordance with studies showing that LPCAT3 prefers polyunsaturated fatty acid acyl CoAs (18:2-acyl-CoA and 20:4-acyl-CoA) as substrates leading to the formation of arachidonoyl and linoleoyl glycerophospholipids (Kazachkov et al., 2008), as observed in the present study. The fatty acyl chains at the sn-2 position of phospholipids are hydrolyzed by phospholipase A2. However, our data show that LXR activation has no impact on cPLA2 gene expression and phospholipase A activity in aortic endothelial cells, suggesting an accumulation of MUFAs and PUFAs in the membrane and therefore limiting the free pool of FAs. Overall, our data show that LXR activation increases the expression of LPCAT3 in aortic endothelial cells, which could increase the incorporation of arachidonic acid into glycerophospholipids, thereby reducing levels of saturated fatty acids.

The preference of LPCAT3 for PUFAs as substrates prompted us to investigate whether LXRs would also regulate PUFA synthesis. FAs generated through *de novo* synthesis make up the pool of intracellular FAs that can be used by LPCAT3 and PLA2 for the synthesis of glycerophospholipids and eicosanoids release. We show that LXR activation increases the conversion rate of n-3 and n-6 FA precursors into AA, EPA and DHA in human aortic endothelial cells which was confirmed by the upregulation of the expression of all the key lipogenic genes. Accumulation of PUFAs has been involved in regulating the activity of ACSL3 itself and protecting against palmitic acid lipotoxicity (Ishida et al., 2015). Enhanced SCD1 activity by LXR agonist also protected against saturated FA lipotoxicity (Peter et al., 2008). Upregulation of desaturase activity by LXR facilitates the desaturation of saturated FAs and prevents the downstream effects of lipotoxicity. As expected also, LXR activation increase MUFAs in neutral and polar FAs fraction. LXR activation has been reported to induce the preferential formation of C16 and C18 MUFAs belonging to the n-7 or n-9 families, resulting in an increased of MUFAs in macrophages and ECs (Wang et al., 2004; Peter et al., 2008; Paton and Ntambi, 2009; Varin et al., 2015). This LXR-driven synthesis of MUFAs may be important to prevent the lipotoxicity induced by the intracellular accumulation of saturated fatty acids. It also enables the synthesis of PUFAs that are potent ligands for transcription factors, such as PPARs (Schleicher et al., 2008). Desaturase Δ5 and Δ6 (FADS1 and FADS2) are required for PUFA synthesis (Sprecher, 2000). Our results show that gene expression of FADS1 and FADS2 was upregulated by LXRs in human aortic endothelial cells. This is in accordance with the transcriptional profiling analysis in normal human epidermal keratinocytes, which identified these desaturases as LXR-responsive genes due to the presence of an LXR-RXR binding site in their upstream region (Shen et al., 2011). However, we cannot exclude a possible direct action of sterol regulatory element binding proteins (SREBPs) as expression of the enzymes involved in FAs biosynthesis is also controlled by SREBPs, which in turn are regulated by LXRs (Amemiya-Kudo et al., 2002). LXR agonist did not change neither mRNA expression of cPLA2 nor the phospholipase A activity. cPLA2 is selective for the hydrolysis of phospholipids and especially phosphatidylcholine containing AA in the sn-2 position compared to other FAs. It can also hydrolyze phospholipids containing HETEs or HpETEs in this position. It has been shown that linoleic acid could increase cPLA2 activity and the release of arachidonic acid in hepatocytes (Suh et al., 2008) but we did not observed any accumulation of linoleic acid upon LXR activation suggesting a minor role of PLA2 in the free MUFAs/PUFAs generation. Therefore, arachidonic acid accumulation observed in Figure 1 is mainly due to the desaturation reaction as measured in Figure 5. In Figure 1A, we can see a small but significant accumulation of C16:1n-7 and C18:1n-7 but no modification in the other MUFAs. C16:1n-9, C18:1n-9 and C20:1n-9 were slightly predominant in untreated cells compared to treated cells (even if not statistically different) and therefore the sum of MUFAs in total FAs fraction would smooth the differences between the two group of cells (Figure 1B). The sum of SFA and MUFA were significantly modulated in polar and neutral FAs fractions, but these differences were not significant when looked at the larger scale i.e., total FAs (Figures 1B,D,F). Some hypothesis might explain these discrepancies. In a technical point of view, as expected analysis at the larger scale did not allow to recover the same yield of FAs (these was observed by using internal standard). A higher yield was observed for neutral and polars FAs which allows to precisely *i*, discriminate the different species of FAs and ii, quantify in a more accurate way FAs especially those present in low quantity. We could also make the hypothesis that LXR treatment may stimulate the formation of free SFA and MUFAs through phospholipase A activation after LXR agonist treatment. It has been shown that LXR agonist stimulated secreted sPLA2-IIA promoter and sPLA2 activity in oligodendrocyte cell line (Trousson et al., 2009) but the effect seems to be dependent on LXRβ which was not modulated in our condition. However, we cannot exclude such mechanism in our condition. Equally noteworthy that the distribution of SFA and MUFAs is different between polar and neutral lipids. Indeed, C18:1n-9 decrease after LXR activation in neutral lipids fraction but not in polars fraction and the overall sum would equalize the quantity of this FAs in total FAs pool between treated and control condition. The same effect is observed for the palmitic acid. Therefore in our condition, the sum of SFAs and MUFAs in total FAs as observed in Figure 1B, may underestimate the effect of LXR treatment on these FAs and underlines the need to analyses the subclasses of FAs i. e., the polar and neutral FAs fractions. Other additional analysis of the fate of free FAs after LXR agonist treatment remain to be identified.

Increased PUFA synthesis in aortic endothelial cells by LXRs may have significant biological effects on endothelial function. PUFA metabolites like eicosanoids and other oxygenated products have been known to play a role in inflammation and cardiovascular disease (Wymann and Schneiter, 2008). Our data show that LXR activation decreases the number of lipoxygenase-derived lipids. ECs are able to generate prostaglandins as well as mono and di-hydroxyeicosatetraenoic acids (di-HETEs) from linoleic acid and arachidonic acid. Prostacyclin is the main product from arachidonic metabolism via cyclooxygenase activity. Our data show that LXR activation does not change the synthesis of prostaglandins, especially PGI2. This could be due to the absence of cPLA2 upregulation leading to the accumulation of arachidonic acid (AA) at the membrane interface. This is consistent with the decreased production of 15-HETE from AA that was observed. The exact roles of 15-HETE is not entirely clear, in part because 15 lipoxygenase also generates potent anti-inflammatory mediators, including AA-derived lipoxins and DHA-derived protectins and D-series resolvins (Powell and Rokach, 2015). Nevertheless, 15-HETE has been shown to be an endogenous competitive inhibitor of vascular cyclooxygenase that inhibits the release of prostacyclin, and plays a role in the promotion of EC migration (Setty and Stuart, 1986). 5-lipoxygenase metabolites of arachidonic acid, 5-HETE, stimulates human microvascular endothelial cell DNA synthesis and cell growth (Zeng et al., 2002). Therefore, LXR activation, leading to increased 5-HETE and decreased 15-HETE levels in ECs, may have an impact on angiogenesis and prostacyclin release. Our data show that LXR activation decreases the synthesis of linoleic acid metabolites derived from lipoxygenase activity. Linoleic acid metabolites 13 and 9 - HODE reduce endothelial PGI2 production, and may also regulate platelet interaction with the endothelium (Kaduce et al., 1989). Endothelium is a key component in the physiopathology of sickle cell disease (Wagner and Frenette, 2008). Little is known about lipids metabolism in endothelium in sickle cell disease context. While the activation of LXR signaling modulates the lipid metabolism in endothelial cells, we did not observe a significant alteration of the expression of markers of inflammation. It is know that LXR is an important mediator of endothelial senescence and act as anti-atherosclerosis (Zhu et al., 2008). More analysis are however needed to investigate the role of LXR in sickle cell disease. Our data suggest that by modulating fatty acid membrane composition, LXR activity could be linked to the release of lipid metabolites involved in endothelial function.

In summary, our data highlight that LXR activation increases the PUFA synthesis and esterification in membrane phospholipids, leading to the modulation of prostaglandin release. The data do not exclude a possible indirect effect of LXRs via SREBPs, however it has been shown that most of the genes that code for PUFA synthesis enzymes have an LXR binding site, and therefore support a determinant role of LXRs in lipid metabolism. It has been shown that LXRs are an important regulator of angiogenesis that require a highly dynamic membrane. Our data suggest a mechanical function of LXRs, in which LXRs could regulate angiogenesis by modulating the FA composition of EC membranes. Therefore, it would be insightful to evaluate the interaction between PUFA synthesis and LXs activation in a context of pathophysiological angiogenesis. Our results show that LXRs play a pivotal role in the metabolism of FAs into EC membranes and may serve as a novel target for treating EC dysfunction that involves lipid metabolism.

## Data Availability

The raw data supporting the conclusion of this article will be made available by the authors, without undue reservation.

## Abbreviations

AA, arachidonic acid; ALA, alpha linolenic acid; ECs, endothelial cells; EPA, eicosapentaenoic acid; DHA, docosahexaenoic acid; FAs, fatty acids; LXRs, liver X receptors; LPCAT3, lysophosphatidylcholine acyltransferase 3; MUFAs, monounsaturated fatty acids; PUFAs, polyunsaturated fatty acids.
